# Epiploic Appendagitis Mimicking Recurrent Diverticulitis

**DOI:** 10.1155/2018/1924067

**Published:** 2018-04-11

**Authors:** R. Plummer, Y. Sekigami, Lilian Chen, James Yoo

**Affiliations:** Department of Surgery, Tufts Medical Center, 800 Washington Street, Boston, MA 02111, USA

## Abstract

Here, we report a case in which a patient with an extensive history of diverticulitis of the sigmoid colon presented with left lower quadrant abdominal pain similar to her previous episodes of diverticulitis. An initial diagnosis of diverticulitis was made based on her history and exam, intravenous antibiotics were given, and an elective surgical resection was considered. However, a subsequent CT scan revealed epiploic appendagitis with no evidence of diverticulitis. Though uncommon, in patients with a history of recurrent diverticulitis, alternative causes of left lower quadrant abdominal pain such as epiploic appendagitis should be considered as this may alter future treatment decisions.

## 1. Introduction

Epiploic appendagitis is a relatively rare inflammatory condition of the fat-filled serosal outpouchings of the colon resulting from the obstruction of blood flow within the tissue [1, 2]. The minimal vasculature and pedunculated shape predisposes the appendages to circulatory obstructions through either spontaneous thromboses or torsion of the appendage [3, 4]. Inflammation is most frequently reported in the sigmoid colon and ileocecal region where larger appendages are commonly observed [5]. As a consequence, the associated abdominal pain of the lower quadrants may mimic that of diverticulitis and appendicitis [6]. Definitive diagnosis of epiploic appendagitis requires radiologic evaluation by ultrasound or CT scan [7–9]. Here, we describe a case of a patient with a history of recurrent episodes of diverticulitis in the sigmoid colon who presented with tenderness in the left lower quadrant of the abdomen and was found to have epiploic appendagitis.

## 2. Case Presentation

The patient is a 67-year-old female with a history of hypogammaglobulinemia on IVIG infusions, asthma, breast cancer, abdominal aortic aneurysm status post repair, and recurrent diverticulitis who presented with a one-day history of worsening abdominal pain associated with loose bowel movements. The pain began in the morning and worsened throughout the day, prompting her to be seen by her primary care physician. A diagnosis of recurrent diverticulitis was made, and the patient was transferred to our institution for further management. Her last episode of diverticulitis confirmed by CT scan occurred two months prior, for which she was hospitalized with eventual resolution of her symptoms. The patient has had a total of three prior episodes of diverticulitis, two of which required hospitalization. She reported that the present pain was similar to her last episode.

Her exam on admission was notable for mild abdominal distention with tenderness to palpation in the left lower quadrant without rebound tenderness or guarding. Her WBC count was normal. She was managed conservatively with bowel rest and IV antibiotics. Given the recurrent nature of her diverticulitis, elective surgical resection was discussed.

**Figure 1:**
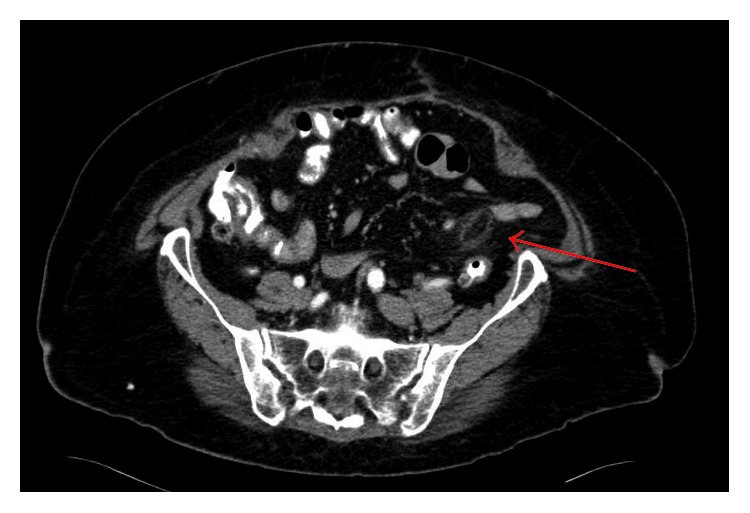
CT findings consistent with epiploic appendagitis including an ovoid fat density structure with surrounding peripheral inflammatory change (red arrow).

Over the next 24 hours, she reported persistent symptoms and a CT scan was obtained, which demonstrated colonic diverticulosis without definite evidence of diverticulitis. An ovoid fat density structure with surrounding peripheral inflammatory change was noted in the left lower quadrant adjacent to a loop of descending colon, consistent with epiploic appendagitis (Figure 1).

Her antibiotics were ultimately discontinued, and she was discharged home. The patient was seen for follow-up one month after discharge and she reported feeling well.

## 3. Discussion

Epiploic appendagitis is an uncommon condition involving inflammation of epiploic appendages resulting from obstruction of blood flow within the tissue. These small projections of adipose tissue are often found in two rows adjacent to the anterior and posterolateral taenia coli, totaling 50–100 in most adults [1, 2]. Each appendage averages 3 cm in length, although size and shape may vary greatly with larger appendages appearing near the sigmoid colon. The nutrient status may affect the size of the appendages, as they tend to be larger in obese individuals and those who recently lost weight [1, 4]. Each appendage is supplied by one or two arteries which branch from the vasa recta longa of the colon and has one draining vein. Although no consensus has been reached on the role of the appendages, it is proposed that they may serve a protective role for the intestinal vessels during times of distension or collapse of the colon [1]. Men appear to be more susceptible to epiploic appendagitis than women [10, 11]. Strenuous exercise and obesity may also increase the risk of developing the condition [12].

The characteristic ischemia and necrosis of the epiploic appendages appear to be a consequence of its minimal vasculature and pedunculated shape. These features predispose the appendages to infarctions and physical manipulations that may completely obstruct circulation [3, 4]. A majority of cases appear to be caused by torsion, leading to ischemia and aseptic necrosis [1, 12, 13]. Other common causes include spontaneous venous thromboses that occlude the single vessel that allow venous return from the appendage [5]. While appendagitis is seen throughout the colon, the majority of cases are observed in the sigmoid colon and ileocecal region [5], most likely due to the increased concentration of larger appendages that are more prone to torsion.

Most patients with epiploic appendagitis present with lower abdominal pain, with a majority in the lower left quadrant. Physical examination reveals associated tenderness without the presence of masses. The pain is generally described as being constant, dull, localized, and nonmigratory. These patients are typically afebrile and deny anorexia, nausea, vomiting, and diarrhea. Lab tests are generally normal, although some patients may demonstrate slightly elevated CRP and leukocytosis [10, 11, 13]. Similarly, our patient presented with left lower quadrant tenderness and minimal additional symptoms. Her WBC count was normal, in accordance with the aseptic inflammation that is typical in patients with epiploic appendagitis.

Diagnosis of epiploic appendagitis is made challenging by the lack of pathognomonic clinical features and should therefore be considered as a potential diagnosis of exclusion [13]. The localization of lower quadrant pain may mimic other more common conditions leading to misdiagnoses. Common sites of epiploic appendagitis, including the ileocecal area and sigmoid colon, produce similar localization of pain typical in those with appendicitis and diverticulitis, respectively [6]. Therefore, preoperative diagnoses should be followed by either computed tomography (CT) or ultrasound (US). With an ultrasound, hyperechoic noncompressible ovoid masses with a hypoechoic ring are found at the site of tenderness [7, 8]. Lack of blood flow resulting from torsion or infarction is also observable through Doppler studies [14]. On CT scan, epiploic appendagitis can be detected as an ovoid structure with a fatty mass circumscribed by inflammatory changes, as observed in our patient. Narrowing of the base of the appendage, consistent with the applied torsion, may also be observed in nonthrombotic cases. Central high density foci may also be observable in some cases, most likely visualizing a thrombosed vessel within the appendage [9].

Epiploic appendagitis is generally considered a self-limiting condition in which patients typically recover within one to four weeks with conservative pain management [6, 10]. Surgical ligation of the appendage may be necessary if patients fail to improve with conservative treatments or if appendagitis becomes recurrent [8, 10, 11, 13]. With a one-day history of pain in our patient, there were minimal concerns of infection from prolonged inflammation or extensive necrosis, and we therefore pursued conservative management. The patient's symptoms resolved by about a month.

## 4. Conclusion

The low incidence and lack of pathognomonic features of epiploic appendagitis make it an easily missed diagnosis. Because this condition shares many symptomatic characteristics with diverticulitis and appendicitis, radiologic studies are crucial for differentiating it from other more common abdominal conditions.
